# Nurse Expertise: A Critical Resource in the COVID-19 Pandemic Response

**DOI:** 10.5334/aogh.2898

**Published:** 2020-05-11

**Authors:** Patricia Nayna Schwerdtle, Clifford J. Connell, Susan Lee, Virginia Plummer, Philip L. Russo, Ruth Endacott, Lisa Kuhn

**Affiliations:** 1 Nursing and Midwifery, Faculty Medicine, Nursing and Health Sciences, Monash University, AU; 2 Heidelberg Institute of Global Health, Heidelberg University, DE; 3 Federation University, AU; 4 Department of Nursing Research, Cabrini Institute, Malvern, AU; 5 University of Plymouth/Royal Devon and Exeter Hospital Clinical School, Exeter, UK; 6 Monash Emergency Research Collaborative, Monash Health, Clayton, AU

## Introduction

Across the world, from our unique place within multi-disciplinary health teams, nurses are preparing for, working to overcome, or recovering from the first waves of the worst pandemic humanity has seen in a century. While in the media, nurses are predominantly depicted providing direct patient care; we are also well represented at every level of pandemic response from advising governments to leading research, coordinating public health teams, and strategizing humanitarian responses to COVID-19. Nurses comprise the largest component of the health workforce, playing a key role in developing practice, and preserving the core values of health systems globally. Through the agency of sound expertise and credo, nurses underwrite best practice and advocate for health equity. During this pandemic, nurses continue to research, inform policy, and where appropriate, effect change to the full extent of their expertise for the benefit of public health [1,2]. Nurse expertise in infection prevention and control, critical care, palliative care, and public health will constitute the difference between the success and failure of global health systems to hold or collapse, and the preservation or loss of countless lives. Central to all of these roles is our adaptability and our capacity to preserve human dignity and ease suffering. Nurses see and practice healthcare through a different lens to other health professions and are often described as the linchpin of health systems. We are the group of healthcare professionals with the closest and most constant proximity to patients and are often exposed to danger and moral dilemmas, facing impossible choices in the context of overwhelming need amid resource constraints. Nurses are uniquely placed to convene multi-disciplinary health professional teams for patient safety and wellbeing [3]. Nursing care is the greatest investment made in healthcare and accordingly has the greatest impact on patient outcomes. With this in mind, we face an extraordinary challenge to respond to a situation that is without precedent, highly uncertain, and changing constantly in countries worldwide. As leading nurse scholars and clinicians, we focus here on critical aspects of the pandemic response, moving from populations to systems, to people, incorporating both curative and palliative considerations. We provide practical, solution-based perspectives in a humble effort to help mitigate health risks and to strengthen the health service response.

## Populations

### Protect health systems

Health systems represent each country’s source of hope in disaster. In our globalized world, health systems are inter-dependent. For example, when one system successfully ‘flattens the curve’ (and thereby spreads the demand for healthcare over time) that jurisdiction may have the capacity to accept patients from other states or countries or to share PPE and other key resources. This is evidenced by WHO’s efforts to improve the availability of critical resources for heavily affected countries through a collaboration with governments and industry around the world [4]. Individual health systems are a part of one worldwide health system serving a global community. Under the weight of COVID-19, some elements of this system may fail, with devastating consequences that extend far beyond COVID-19-related morbidity and mortality. To date, some health systems have been agile across many countries and have reacted radically and deftly in response to the pandemic threat. For instance, routine elective surgery procedures have been postponed to maintain resource availability for COVID-19, and we have seen some innovative task-shifting and vital equipment sharing to meet needs. Further, services have been split into COVID-19 and non-COVID components, with mechanisms to identify and transfer patients and staff between them to reduce cross-contamination [5]. Just as it is estimated that many more people suffer in the aftermath of a disaster than from the event itself, many more people will die or suffer non-COVID health issues when systems are overwhelmed or collapse. This is particularly the case in low-resource countries [6]. Whilst all elements of health systems will be affected by COVID-19, essential primary, secondary, and tertiary services must be maintained throughout the pandemic.

### Contain and mitigate in parallel

Whilst the impact of COVID-19 on health systems to date is unprecedented, we do have tested and effective public health interventions that can be boosted by technology and innovation, such as using mobile phone technology to improve case tracing [7]. Although there is no one-size-fits-all solution, various countries combine containment strategies with different degrees of i) mitigation, to reduce transmission, some aiming for herd immunity, or ii) suppression, to interrupt transmission until a vaccine becomes available [8]. Containment strategies that aim to test (identify), isolate (cases), trace, and quarantine (contacts) are important aspects of both strategies but are not enough to adequately protect the community. Societies will be affected by both the pandemic itself and the adverse impacts of mitigation and suppression interventions that can lead to increased unemployment and poverty, amplified domestic violence, mental health issues, and social unrest [9]. We recognise and are concerned that these impacts may be particularly severe in low-resource regions that have fragile health systems, a lack of critical care capabilities, and absent social safety nets to buffer the impacts of lockdowns [10]. Continued widespread testing allows for a more nuanced approach to restrictions on people’s livelihoods and freedoms, resulting in a more sustainable public response, reducing the collateral damage that occurs during prolonged lockdowns. Countries can learn from the experiences of others, tailoring responses to their unique health, social, and economic environments and capabilities. In all countries, there is a pressing need for nurses and all health professionals to advocate and intervene where appropriate for people who are in vulnerable situations, such as organised advocacy efforts calling for governments to evacuate refugee detention facilities and to include migrants and refugees in health and social protection schemes. Nurses worldwide have had to change their model of care to a population-based model with the rapid onset of this pandemic. Nurses everywhere are demonstrating innovation and leadership in their positive approach to keeping communities, patients, and themselves safe. There is a need to prioritize the vulnerable from a global perspective (refugees and asylum seekers), as well as from a national perspective to account for inequity within societies, including those people living in poverty, homeless, or imprisoned.

### Pandemic models of care

Health systems that incorporate models of care that are centred on hospitals are common throughout the world, particularly in urban centres. Such systems are focused on the care of individuals rather than what is best for the community. They are well designed for dealing with expected disease burden and relatively small numbers of highly complicated, critical illnesses, but they are particularly vulnerable to collapse in pandemics [11], in which case a population-based model of care is required. Robust health services also provide decentralized healthcare delivery in a range of settings, such as in private homes, outreach services, and community health programs. They enable the ministering of healthcare to populations to prevent disease, rather than to individual patients in need of complex and resource-intensive healthcare.

As in medicine, public health is a core element of nursing education. As such, nurses play key roles in many decentralized community health programs and population-level services. Health systems with a strong community focus that value preventive interventions and integrate community engagement and empowerment may have an advantage in pandemic response compared to the more traditional biomedical, hospital-centric systems. In community-centred settings, the acutely and chronically unwell do not need to congregate together over extended periods to receive healthcare in a single setting. Further, systems that enable outreach and home care with monitoring and referral for mild to moderate cases of COVID-19 have the potential to relieve pressure on hospital critical care services and avert overwhelming the inpatient, hospital-based system. The challenge will be to adapt systems and processes quickly to shift from patient- to population-centred care.

## Systems

### The new COVID-19 health workforce

With approximately 5% of infected patients needing critical care and 30% of those requiring admission in an intensive care unit (ICU) [12], COVID-19 is placing immense pressure on critical care services everywhere. Many health systems are adapting rapidly by preparing extraordinary and new critical care workforce models [13]. We are asking former and future colleagues, retiree nurses, general nurses, and undergraduate nursing students alike to step into roles where the requisite knowledge, clinical skills, and experience need to be refreshed and re-learned or are yet to be acquired. Experienced critical care nurses familiar with current practice and technology will supervise large numbers of returning and new nurses in ICUs, many of whom will be anxious about the extra responsibility and implementing unfamiliar practices such as managing ventilated patients in the prone position, who are also highly infectious. Nurse practitioners and nurses in other advanced-care roles are likely to play a central role in training and mentoring. This new model of nursing care delivery calls for rapid critical care teaching and learning to prepare the new nursing workforce in a swift but robust manner to minimize risk, optimize patient safety, and reduce the burden of supervision.

### Multiple sources of information, expert sources of truth

Nurses with qualifications in infectious diseases, disaster management, public health, and epidemiology are ideally placed to provide expert advice at governmental levels [14]. Ironically, in pandemic situations, the overwhelming demands for their expertise at the local level can muffle their voices further afield. Several issues interfere with operationalizing infection control and prevention guidelines including conflicting advice from various organisations, professional colleges and societies, and jurisdictional health departments. Health professionals can feel overwhelmed by the volume of evidence that seems to be constantly changing [15]. Nurses play a key role in cutting through the information overload and disseminating evidence-based knowledge wherever they are. In a rapidly evolving situation mixed with a profuse amount of new data and commentary, much of which is not based on a full appraisal of the evidence, it is extremely challenging for nurses and other health professionals to source the highest-level evidence to inform their practice. The challenge for infection prevention nurses has never been so great. Suddenly, infection prevention is foremost in everyone’s mind. The marshalling of all health professionals in a response united in collaboration with infectious disease experts will determine the trajectory of this pandemic and serve as a model for future challenges.

### Finite PPE and prioritization

Despite a large number of nurses and other health professionals having contracted COVID-19 in many countries, reporting occupational prevalence at the national and global levels is inadequate [16]. When nurses and other health professionals contract SARS-CoV-2, the virus that causes COVID-19, at work, infection prevention programs are immediately drawn into the spotlight. There is a finite supply of PPE and occasional confusion regarding it’s use due to changing and inconsistent PPE recommendations. This leads to increased anxiety among health professionals made worse by insecure PPE supplies. Inappropriate use of PPE and incorrect removal (‘doffing’) increases the risk of self-contamination, yet health professional training has struggled to keep pace with the pandemic. Prioritizing the use of PPE to high-risk areas is a constant challenge and needs to be informed by local epidemiology. Nurses play a key role in optimizing PPE availability through minimising the need for PPE (e.g. leading health information hotlines and triage), using PPE appropriately (delivering staff education), and coordinating PPE supply chains [17]. Nurses have also led innovative efforts to reduce PPE use such as streamlining patient assessment systems to reduce staff risk [18]. After the current crisis abates, nurses and other health professionals will need to work with government, health services, and regulatory bodies to ensure that future stores of PPE are retained and fit for purpose and that PPE use is enveloped into the regularly evaluated core competencies of all healthcare workers [19].

## People

### Family-centred care

Standard practices for keeping family and friends in touch with and informed of patients’ conditions have been hampered by physical distancing and isolation measures, Innovative and timely use of communication technologies are urgently needed. Nurses and other health professionals have reported using their phones to enable patients to connect with loved ones, sometimes just before endotracheal intubation and mechanical ventilation from which many will not recover. Nurses are utilising internet communication platforms to engage family members, inviting them to virtual bedsides to communicate with patients, hear familiar voices (even when unconscious), and involve loved ones in decisions about their care. Sudden and overwhelming illness may have overtaken the discussions relatives may have otherwise had with patients about their wishes for future care. The urgency of decisions and pressure on staff and services may impede considered discussion. However, the existence of advance care plans and the availability of alternate decision-makers are important to determine. Taking time to ask about these may free family members and health professionals from the burden of making difficult decisions about treatments and reassure them about what the patient would have wanted. Nurses who are experienced in caring for people who die and more experienced nurses are reported to be comfortable in raising advance care planning questions with patients and family members [20]. Models of care for COVID-19 patients should include nurses who are experienced and comfortable with inpatient and family education to establish advance directives.

### Self-care and wellbeing

Nurses and many health colleagues are facing unprecedented workloads and profound sadness whilst making grave and consequential decisions not usually required in times outside of war and disaster. Where the pandemic has peaked, many are working for weeks without a break. Nurses are the go-to person for family and friends at work and home: there is no ‘off’ button. In addition to the worry caused by having to work without adequate stocks of PPE, many nurses and other health professionals fear infecting their loved ones and so are self-isolating at home or moving away from their support systems. During times of acute clinical need, nurses and other health professionals often continue working and defer grief until after an event has passed, putting them at heightened risk of burnout and post-traumatic stress disorder (PTSD) [21]. The risk of burnout, compassion fatigue, and PTSD are further compounded by engaging nurses without critical care qualifications in critical care environments [22]. Mental health sequelae from experiences of epidemics constitute an ‘emergency within an emergency’ [23] and will need to be mitigated in the months following the crisis [24].

This pandemic will require nurses and other health professionals to face moral dilemmas for which there is no rule book. Now, more than ever, it is time for each country’s healthcare leaders and policymakers to deliver and sustain genuine measures to fortify the welfare of nurses, other health professionals, and their families, who are forced to make intensely complex decisions, often without counsel or debrief. Health care leaders are well positioned to facilitate briefing, debriefing and psychological support through skilled communication that is unambiguous and compassionate. To avert a future workforce crisis due to compassion fatigue and burnout, these support systems will need to continue well beyond the COVID-19 crisis.

## Conclusion

COVID-19 will test our ability to think, learn, adapt, and act as a global health workforce, and our success will depend on our solidarity within teams, communities, nations and globally. As we face an intimidating and uncertain future with the worst yet to come for some countries, it is difficult to imagine what lies beyond. However, we must see the aftermath as an opportunity to redefine many aspects of healthcare that have been problematic for some time. We expect nurses to take a prominent role in these discussions and the design of new ways of working. If nurses and all health professionals continue to collaboratively examine the utility, equity, and implementation of healthcare through this period, public health policymakers can be confident that decisions arising from this pandemic are wholly informed by all key healthcare stakeholders. This is a complex and difficult task that requires a level of collaboration that health workers have long sought but have yet to achieve. It is, however, self-evident that a privation of the expertise and oversight of one of healthcare’s most ubiquitous and essential groups will yield a dangerously incomplete response to this crisis. Further, the post-pandemic evaluation should not be limited to the health sector that does not stand alone against threats of this scale, now or in the future. The underlying drivers, including environmental considerations of the pandemic and barriers to an optimal response, must be thoroughly examined.

We draw energy and inspiration from the renewed support and admiration for nurses and the broader health workforce that we see through applause from balconies, rooftops, and front gardens around the world. We have outlined a number of the challenges and have offered some solutions at the levels of population, systems, and people from a nursing perspective, yet we acknowledge the importance of connecting professions, services, and sectors together at this time, with patients and populations at the centre.

**Caveat:** This editorial reflects the views of the authors at the time of writing and the particular stage of pandemic evolution.

## Author Information

Patricia Nayna Schwerdtle RN, MPH, PGC-HPE, PGC-Crit.Care, BASci.HP, BN. Global Health Academic and Adjunct Academic at Monash University (Australia). PhD Candidate at Heidelberg Institute of Global Health, Heidelberg University (Germany). Director on the Board of Medecins Sans Frontieres, Australia. patricia.schwerdtle@monash.edu. ORCiD: 0000-0002-3045-3145.

Clifford J. Connell RN, PhD, BN (Hons), Grad cert. critical care (ED), Grad Cert Health Prof Ed. Monash Nursing and Midwifery Postdoctoral Research Fellow Monash Nursing and Midwifery, Monash University. clifford.connell@monash.edu. ORCiD: 0000-0002-2765-6355.

Susan Lee RN, PhD, MBioeth, BAppSci (NurseEd), DipAppSci(Nurs) is the teaching and research academic at Monash University (Australia) with a particular focus on palliative and end of life care, decision making and ethics. Susan.Lee@monash.edu. ORCiD: 0000-0003-4288-7394.

Virginia Plummer RN, PhD, MSc (Health Policy & Management), Grad Dip Health Admin, GCHPE, Grad Cert Emergency Health (Disaster Prep & Management) Crit Care Cert, Mid Cert, BN) FACN, FACHSM, Member WADEM. Adjunct Associate Professor Nursing and Midwifery at Monash University and Adjunct Professor Nursing and Midwifery at Federation University Australia. ORCiD: 0000-0003-3214-6904.

Philip L. Russo RN, PhD, MClinEpid, BN, CICP-E, MACN, FACIPC. Director, Cabrini Monash University Department of Nursing Research. Associate Professor Nursing and Midwifery at Monash University. President of the Australasian College of Infection Prevention and Control. ORCiD: 0000-0003-3822-0554.

Ruth Endacott, RN, PhD (Med Sci), MA (Res Meth), DipN, Professor of Critical care Nursing, Monash University. Professor/Director of the University of Plymouth/Royal Devon and Exeter Hospital Clinical School, UK. ORCiD ID: 0000-0002-4352-4600.

Lisa Kuhn, RN, PhD, MHSc, GDNur, EmergCert, DipAppSc(Nur), MACN, MACCCN, FCENA. Associate Professor of Emergency Nursing, Monash Nursing and Midwifery, and Chair (Nursing) Monash Emergency Research Collaborative, Monash Health. Lisa.Kuhn@monash.edu. ORCiD: 0000-0002-2421-2003.
